# Effectiveness of a problem-solving based intervention to prolong the working life of ageing workers

**DOI:** 10.1186/s12889-015-1410-5

**Published:** 2015-02-04

**Authors:** Wendy Koolhaas, Johan W Groothoff, Michiel R de Boer, Jac JL van der Klink, Sandra Brouwer

**Affiliations:** Department of Health Sciences, Community and Occupational Medicine, University Medical Center Groningen, University of Groningen, Antonius Deusinglaan 1, FA10, Building 3217, room 621, 9713 AV, Groningen, The Netherlands; Department of Health Sciences, VU University, Amsterdam, The Netherlands

**Keywords:** Aging workforce, Intervention study, Ageing workers, Problem-solving approach, Solution focused, Work ability

## Abstract

**Background:**

An ageing workforce combined with increasing health problems in ageing workers implies the importance of evidence-based interventions to enhance sustainable employability. The aim of this study is to evaluate the effectiveness of the ‘Staying healthy at work’ problem-solving based intervention compared to business as usual.

**Methods:**

This study was designed as a quasi-experimental trial with a one-year follow-up. Measurements were performed at baseline, three and twelve months. The problem-solving based intervention provides a strategy for increasing the awareness of ageing workers of their role and responsibility in living sustainable, healthy working lives. The primary outcomes were work ability, vitality and productivity. Secondary outcomes were perceived fatigue, psychosocial work characteristics, work attitude, self-efficacy and work engagement.

**Results:**

Analyses were performed on the 64 workers in the intervention and 61 workers from the business as usual group. No effects on productivity (OR = 0.83, 95% CI 0.23-3.00) and adverse effects on work ability (B = −1.33, 95% CI −2.45 to −0.20) and vitality (OR = 0.10, 95% CI 0.02-0.46) were found. Positive results were found for the work attitude secondary outcome (B = 5.29, 95% CI −9.59 to −0.99), the self-efficacy persistence subscale (B = 1.45, 95% CI 0.43-2.48) and the skill discretion subscale of the Job Content Questionnaire (B = 1.78, 95% CI 0.74-2.83).

**Conclusion:**

The results of the problem-solving intervention showed no positive effects on the three outcome measures compared to business as usual. However, effectiveness was shown on three of the secondary outcome measures, i.e. work attitude, self-efficacy and skill discretion. We presume that the lack of positive effects on primary outcomes is due to programme failure and not to theory failure.

**Trial registration:**

The trial is registered with the Dutch Trial Register under number NTR2270.

## Background

The age of retirement is being pushed back as state benefit systems fail to keep pace with low birth rates and rising life expectancy [1,2]. This major shift in demographics will force more of the workforce to remain active in the employment market into later life and exerts pressure on society with respect to health, wealth and social insurance systems [3,4]. Ageing is associated with higher sickness absenteeism rates and more disability [5], reduced work ability [6] and decreased productivity [7,8]. Moreover, ageing of the workforce is associated with an increase in the number of workers with chronic health conditions [9-11]. There is evidence that the presence of chronic health problems impacts on work ability [12], work disability [13] and productivity [14] when adjusted for age. The ageing workforce combined with increased health problems in ageing workers implies the importance of evidence-based interventions to enhance sustainable employability [13,15]. To date, this type of intervention is rare.

An important first step in developing strategies and preventive measures aimed at maintaining and enhancing sustainable employability in ageing workers is to get insight into the obstructive and facilitative factors from the workers’ perspective. In a recently published study we gathered the number and type of perceived problems, obstacles, retention factors and support needs of workers aged 45 and older [14]. Workers with different types of self-reported chronic health conditions report significantly more perceived problems due to ageing, obstacles in performing work tasks and support needs to continue their working lives compared to workers without chronic health conditions. However, the type of reported problems, obstacles, retention factors and support needs were diverse but very similar in both groups. These findings suggest that interventions aimed to enhance sustainable employability can be similar for ageing workers regardless of whether they have chronic health conditions. Moreover, it suggests that a preventive intervention to overcome the challenge of an ageing workforce should be able to deal with individually experienced problems and needs.

The cognitive-behavioural process as described by Meichenbaum [16] and D’Zurilla [17] is a possible strategy to help ageing workers deal with these problems, obstacles, retention factors and support needs. Both described a problem-solving approach through which subjects identify effective or adaptive solutions for problematic situations encountered in the course of everyday living. The main goal of a cognitive behavioural approach is to replace maladaptive coping, cognitions, emotions, skills and behaviours with more adaptive ones.

Our intervention, ‘staying healthy at work’, was developed on the basis of the two general, partially independent components of the cognitive behavioural approach ‘problem orientation’ and ‘problem-solving style’ [16,18]. The intervention’s cognitive-behavioural approach could help workers point out a variety of potentially effective solutions for a particular health related problem which affected their sustainable employability. Moreover, this approach increases the probability of selecting the most effective solution from among the various alternatives for workers to continue work participation. This is in line with the cognitive behavioural approach and could help workers learn that they can always influence the impact of a situation by: (1) changing the situation by themselves; (2) mobilizing the support of others; or (3) accepting the situation if it proves to be unchangeable. It contributes to the workers’ belief that they are capable of solving work-related problems and attaining goals, and thereby strengthens their self-efficacy in remaining in work.

The cognitive behavioural approach appears effective for different patient groups of all ages [19-23] and contributes to an earlier return-to-work for pain patients [24]. The problem-solving approach in a workers context facilitates return-to-work and shortens the duration of recurrent sickness absence [25]. The focus in those studies was on workers on sick leave and thus on tertiary prevention, which makes it quite natural that the occupational physician was the appropriate mediator for facilitating those workers. The present study based enhancing sustainable employability of ageing workers on individual arrangements, career development activities and aspirations. For this primary preventive approach the supervisor is the most suitable mediator. Supervisors are most likely to receive the first indications that adjustments are required for workers and are responsible for supporting and facilitating workers [26,27]. Therefore, the ‘staying healthy at work’ intervention trained supervisors in supporting workers to take the necessary actions by means of encouraging self-direction and enhancing knowledge and competences.

The aim of the present study was to evaluate the effectiveness of this problem-solving based intervention in maintaining and enhancing sustainable employability compared to business as usual. In addition, a process evaluation of the intervention was performed to explore which problem-solving approach and programme delivery method was responsible for the results.

## Methods

### Study design and setting

This study was carried out as a quasi-experimental trial. The outcomes were measured at baseline, three and twelve months. A process evaluation was performed at worker level at three and nine months. The Medical Ethics Committee of the University Medical Center Groningen approved the study design, the protocols and the procedures.

### Study population and recruitment

The study was performed at the University Medical Center Groningen (UMCG) and the University of Groningen. The participating UMCG departments were the paediatric and intensive care units (nurses). The University departments which participated were technical services (maintenance and repair), secretarial administrative services (administration), financial economic affairs (policy development, consulting and implementation), human resource advice (policy staff), facility services (cleaning maintenance) and the University Library (management and services). The mean age of the workers was 52.4 (SD 4.9), or 51.7 (SD 4.8) and 52.9 (SD 5.1) from the intervention group and the business as usual group, respectively. Most frequently self-reported chronic health conditions were musculoskeletal diseases (62%), followed by neurological or sensory disease (28%) and mental health conditions (24%).

Participants in this study were selected in a two-step procedure. First, eligible supervisors were invited to participate in the trial. Human Resource Professionals (HRPs) from the two organizations selected the supervisors and thus their workers. The inclusion criteria for potentially participating departments in this study were: a) a higher proportion of workers aged 45 and over than other departments; b) no other intervention studies being performed simultaneously; and c) no planned reorganizations. Supervisors at the departments recommended by the HRPs were invited and informed about the set-up of the intervention by the researcher prior to their decision to participate in this study. Those first to agree to participate in this study were allocated to the intervention group until half the estimated available workers (n = 131) had been allocated. The remaining supervisors and their workers formed the business as usual group. Supervisors willing to participate in the study received information about the process. Recruitment of the supervisors started in June 2009.

In the second step, the eligible workers of supervisors who consented to participate were asked to participate in this study. Workers were informed about this study by their supervisor in November 2009. The workers then received a letter inviting them to participate in the study, describing its aim, content and set-up. Workers on long-term sick leave with no prospect of recovery, or workers who were certain of retiring within a year were excluded from the study. Participation was voluntary and workers were free to leave the study at any time without further consequences. All the workers with the same supervisor followed the same treatment regime, i.e. treatment allocation was at the level of the supervisor. This was done to minimize the probability of contamination [28].

**Figure 1:**
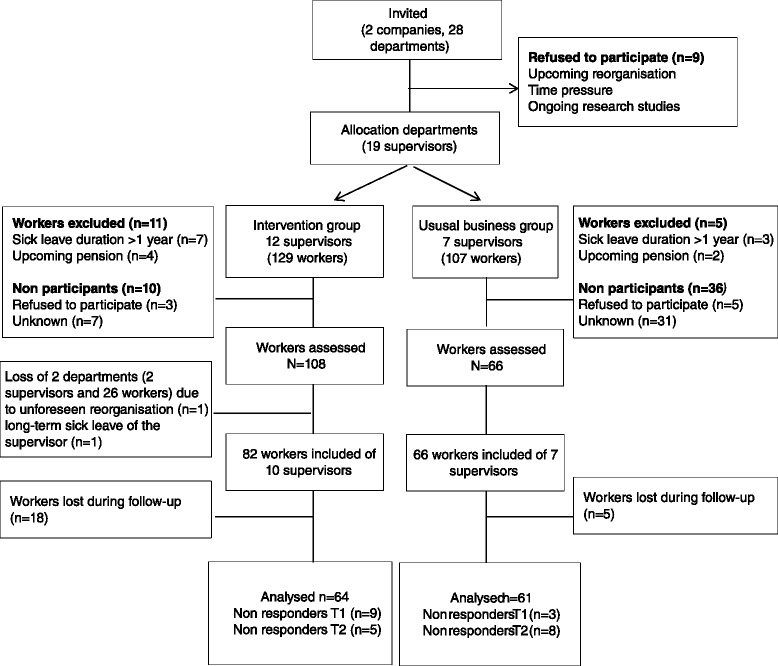
**Flow of the participants throughout the phases of the trial.**

Twenty-eight supervisors, thus 28 departments, were approached to participate in this study. Nine departments declined for reasons such as upcoming reorganization and time pressure. The remaining 19 departments and their supervisors were allocated to either the intervention group (n = 12) or business as usual group (n = 7). Following the allocation of the supervisors, the total of 236 workers were divided between the intervention group (n = 129) or business as usual group (n = 107). An overview of the recruitment flow is presented in Figure 1.

#### Non-participation and loss to follow-up

Workers who did not meet the inclusion criteria were excluded from this study (n = 16). The most common reasons for exclusion were sick leave for longer than 1 year (n = 10) and upcoming retirement (n = 6). In the intervention group, three workers refused to participate, and seven did not participate for unknown reasons. In the business as usual group, 36 workers refused to participate (n = 5) or did not participate for unknown reasons (n = 31). There was no data on the demographics or work-related outcomes of these workers to compare them to the workers participating in this study.

During the intervention, two supervisors from the intervention group were unable to implement the intervention: because of an unforeseen departmental reorganization for one and long-term sickness of the other. Therefore, we lost 26 workers in the intervention group after the baseline measure. For the remaining workers, data for both follow-up measures was missing for 18 and 5 workers in the intervention and business as usual groups, respectively.

### The ‘staying healthy at work’ intervention

The goal of the intervention is to make workers aware of their own responsibility and behaviour in creating a healthy and motivating work environment (e.g. life-long learning) by encouraging them to go through a problem-solving process (performing a problem orientation) to find and implement effective actions and solutions to achieve improvement, life-long learning or to tackle problems which obstruct and deteriorate a sustainable working life (to acquire different problem-solving styles). At the worker level, the intervention comprised three stages: 1) an inventory of work-related problems, needs and the workers’ career and personal development opportunities, including an assessment of the degree to which each issue was amenable to change; 2) a dialogue between worker and supervisor to discuss solutions following a brainstorm format; and 3) making an action plan to plan and implement solutions for a follow-up period next year. The intervention was incorporated into the annual appraisal within the organizations.

The intervention includes a booklet for the workers (stage 1) to prepare for their dialogue (stage 2) and complete their action plan based on solutions chosen and recorded by the workers themselves (stage 3). The supervisors are responsible for recognizing and rewarding excellent performance and providing coaching and for feedback where needed to improve performance shortcomings. Supervisors were also expected to ensure that employees had the tools, resources and training required to carry out their responsibilities successfully. Therefore, they were trained in challenging the workers to reflect on the feasibility of solutions and how to present themselves as a source of support for the worker: not by taking over responsibilities but by strengthening the workers’ autonomy. The first two-hour training course focused on knowledge regarding sustainable employability and on problem-solving techniques. After two weeks the second training event (five hours) was held, which consisted of an active training module in which the problem-solving techniques were practised by role-play with an actor.

### Business as usual

Workers in the business as usual group received their regular annual appraisal.

### Outcome measures

#### Primary outcome variables

*Work ability* was assessed using the Work Ability Index (WAI), a self-administered questionnaire comprising seven items: (i) subjective estimation of current work ability compared with lifetime best (0–100 points); (ii) subjective work ability in relation to both the physical and mental demands of the work (2–10 points); (iii) number of diagnosed diseases (1–7 points); (iv) subjective estimate of work impairment due to diseases (1–6 points); (v) sickness absenteeism during the past year (1–5 points); (vi) own prognosis of work ability after two years (1 or 4 of 7 points); and (vii) psychological resources (enjoyment of daily tasks, activity and personal energy, optimism about the future) (1–4 points) [29]. The WAI is a reliable and valid standardized measure of work ability [30]. Scores range from 7 to 49: higher scores indicate better work ability.

*Vitality* was measured using the single-item vitality scale of the 12-Item Short Form Health Survey (SF12) and its reliability and validity have been documented [31,32]. The item scores were ‘never’, ‘seldom’, ‘sometimes’, ‘mostly’ and ‘always’.

*Productivity* was measured using the quantity scale of the Quality and Quantity (QQ) method measuring productivity loss at work [33]. The QQ provides a reliable and valid tool for measuring quantity and quality of work on a daily basis. Workers were asked to indicate how much work they had done during normal working hours on their last regular working day when compared to normal. The quantity of productivity was measured on a scale from 0 (nothing) to 10 (normal amount) [7]. The outcome was dichotomized into productivity loss (scoring 0–9) and no productivity loss (scoring 10). The quality of productivity was not measured because the quality and quantity question are highly correlated [7].

#### Secondary outcome variables

Secondary outcomes were assessed at baseline and at twelve months follow-up. *Fatigue* was assessed with the eight-item subscale ‘the subjective feeling of fatigue’ from the Checklist of Individual Strength (CIS) [34]. The items were scored on a seven-point Likert scale ranging from ‘Yes, that is true’ to ‘No, that is not true’. Scores were summed to yield possible scores ranging from between 8 and 56; higher scores indicate higher degrees of fatigue. *Psychosocial work characteristics* were measured using the Job Content Questionnaire (JCQ) [35]. The JCQ consists of five subscales: job demands (5 items, range 12–48), decision authority (3 items, range 12–48), skill discretion (6 items, range 12–48), social support from supervisors (4 items, range 4–16) and coworker support (4 items, range 4–16). Each item was rated on a four-point scale from ‘strongly disagree’ to ‘strongly agree’.

*Perceived work attitude* was measured using the Dutch Language version of the Work Involvement Scale (WIS-DLV), which covers six items on a four-point scale (strongly disagree to strongly agree). Higher scores (range 0–100) indicate more positive attitudes towards work [36]. *Self-efficacy* was measured using the standardized Dutch version of the General Self-Efficacy Scale (ALCOS-16) [37], which assesses the subjects’ expectations of their general capacities [38]. The instrument consists of sixteen items assessing the subscales ‘willingness to exert effort in completing the behaviour’ (range 6–30), ‘persistence in the face of adversity’ (range 6–30) and ‘willingness to initiate behaviour’ (range 4–24). The five response categories range from ‘strongly disagree’ to ‘strongly agree’: a higher score on this questionnaire indicates higher self-efficacy. *Work engagement* was measured using mean scores from the short Dutch version of the Utrecht Work Engagement Scale (UWES-9) [39]. The nine items reflect three subscales covering the underlying dimensions of engagement. These subscales – vigour, dedication and absorption (each subscale 3 items) – are scored on a seven-point frequency rating scale ranging from ‘never (0)’ to ‘always/ever’ (6). High mean scores on all scales indicative for work engagement are.

#### Potential confounders

At baseline, data on potential confounders at the worker level was assessed using a questionnaire including age, gender (male/female), education (low = lower vocational education/medium = intermediate secondary or vocational education/high = higher vocational education and university), occupation (executive/secretarial or administrative/policy/management), sector (healthcare/education), shift work (yes/no), duration of current position and years of paid work (0-10/11-20/21-30/31-40/>40 years).

#### Process evaluation at the worker level

The process evaluation was carried out using a questionnaire at worker level to measure whether the intervention was carried out as intended. The extent to which the intervention was delivered as planned, the exposure and the engagement of the workers with the intervention and the workers’ attitude towards the intervention were included based on the process elements as described by Steckler and Linnan [40].

The extent to which the intervention activities were executed as planned was measured by the intervention dose delivered (representing how much of the intervention was delivered to the worker in question), operationalized by variables such as whether the information about the intervention was sent to the workers, whether the planned dialogues between the worker and supervisor occurred, and whether the workers received the booklet to prepare for this dialogue.

The extent to which the workers were exposed to the intervention was operationalized by measuring reach and dose received. Reach was defined as the attendance rate. Dose received represents the extent to which the workers actively engaged with the intervention components (e.g. preparing for the dialogue using the first part of the booklet or drawing up an action plan).

Workers in the intervention group were asked (yes/no) to evaluate the content and the relevance of the information leaflets they received to prepare for the dialogue, to what extent they experienced support from the booklet and whether the focus on the amenability of situations to change motivated them to plan and implement solutions. The quality of the dialogue (rated on a five-point scale from excellent to very poor), the duration of the dialogue (in minutes) and the support they experienced from the supervisor were assessed.

### Statistical analysis

All statistical analyses were carried out at the worker level and according to the intention-to-treat principle. The chi-square test (ordinal and nominal variables) or t-test (mean scores) were used to compare differences on baseline characteristics between the intervention and business as usual group. For the primary outcomes, we performed linear (work ability), ordinal (vitality) and logistic (productivity) multilevel analyses. We had planned to incorporate three levels (supervisor, worker and observation (time)) in all the models. However, random coefficients at the supervisor level did not improve model fit and resulted in the same effect estimates. Therefore, two random effects (worker and observation) were incorporated in the final models. We tested for interactions between the intervention and time to follow-up by incorporating interaction terms in all the multilevel analyses. For the secondary outcomes we performed linear regression analyses. All analyses included adjustment for baseline levels of the outcome, baseline levels of the other primary and secondary outcomes, and for the potential confounders. A detailed description of the sample size analyses is provided elsewhere [41]. The multilevel analyses were performed with MLWin version 2.24. Linear regression analyses were carried out using the statistical package SPSS version 18.0 (SPSS Inc. Released 2009, Chicago: SPSS Inc). A two-tailed p-level of < .05 was considered to indicate statistical significance for all analyses.

## Results

The 49 workers who dropped out after baseline had a lower educational level (p = .003), were more fatigued (p = .008) and reported lower scores on vitality (p = .004) and on the self-efficacy scales willingness to exert effort in completing a behaviour (p = .007) and willingness to initiate behaviour (p = .026).

Data for 125 workers (53%) was available to analyse the effectiveness of the intervention: 64 workers in the intervention group and 61 workers in the business as usual group.

### Baseline characteristics

The baseline characteristics of the workers in the intervention and business as usual group are presented in Table 1. The occupation of 74% of the workers was executive is nature (85% intervention group versus 53% usual business group) and 16% performed an administrative function (respectively 7% intervention group and 26% usual business group). Based on self-report, in both the intervention and usual business group 13% of the workers do not have a chronic health condition. Of the workers in the intervention group reported 34% and 23% reported respectively the presence of one and two chronic health condition compared to respectively 23% and 20% for workers in the usual business group. The percentage of workers with three or more chronic health conditions is higher in the usual business group compared to the intervention group (44% vs 30%). Most reported chronic health condition in the intervention and usual business group were musculoskeletal diseases (respectively 53% and 68%), followed by neurological or sensory diseases (32% and 25% respectively) and mental health conditions (23% for both groups). Significant differences at baseline between the intervention and business as usual group were found for gender, occupation, sector and shift work. The intervention group consisted of higher percentages of males (27% vs. 11%; p = .003), more executive workers (85% vs. 53%) and fewer secretarial workers (6% vs. 26%; p = .011), more healthcare workers (81% vs 66%; p < .05) and more shift workers (77% vs. 54%; p < .001) compared to the business as usual group. Table 2 shows the frequencies and mean scores of the outcome variables at baseline and follow-up. No significant differences were found at baseline between the intervention and business as usual group.

**Table 1:** **Baseline characteristics of the total population, intervention group and business as usual group (N = 125)**

	**All workers**		**Intervention group**		**Usual business group**	
**Characteristics**	**N**	**%**	**N**	**%**	**N**	**%**
Gender (n = 125)						
Female	100	81	46	73	54	89
Male	24	19	17	27	7	11
Education (n = 125)						
Low	21	17	12	19	9	15
Medium	49	40	21	34	28	46
High	53	43	29	47	24	39
Occupation (n = 125)						
Executive	91	74	53	85	38	53
Secretarial or administrative	20	16	4	7	16	26
Policy	7	6	4	7	3	5
Management	5	4	1	1	4	6
Sector (n = 125)						
Health care	92	74	52	81	40	66
Education	33	26	12	19	21	34
Shift work (n = 125)						
Yes	75	62	47	77	28	54
No	47	38	14	23	33	46
Duration current position in years (n = 125)						
0-10	21	17	12	19	19	15
11-20	26	22	12	19	14	24
21-30	36	30	19	31	17	29
31-40	33	27	18	29	15	25
>40	5	4	1	2	4	7
Years paid work (n = 125)						
0-10						
11-20	15	12	8	13	7	12
21-30	46	37	21	34	25	41
31-40	53	43	31	50	22	36
>40	9	7	2	3	7	11

**Table 2:** **Percentages and means of the primary and secondary outcomes at baseline, 3 and 12 months**

	**Intervention group**						**Business as usual group**					
	**Baseline**		**3 months**		**12 months**		**Baseline**		**3 months**		**12 months**	
	**N = 82**		**N = 55**		**N = 59**		**N = 66**		**N = 58**		**N = 53**	
**Primary outcomes**												
Work ability (mean, SD)	39.4	(5.2)	38.9	(5.2)	38.7	(5.2)	38.9	(5.6)	38.9	(5.1)	38.9	(4.6)
Poor (%)	7		4		4		6		0		2	
Moderate (%)	21		22		22		22		31		23	
Good (%)	50		55		56		52		51		58	
Excellent (%)	22		18		18		20		18		17	
Vitality												
Never (%)	0		0		0		0		0		0	
Seldom (%)	2		2		2		0		3		0	
Sometimes (%)	16		20		20		17		16		25	
Mostly (%)	74		70		73		80		71		68	
Always (%)	8		7		5		3		10		8	
No productivity loss (%)	43		42		36		43		44		34	
**Secondary outcomes (mean, (SD))**												
Perceived fatigue	18.40	(9.2)			19.52	(10.4)	19.18	(9.9)			21.55	(11.4)
Job content												
Job demands	31.66	(4.7)			31.71	(5.4)	32.67	(6.6)			31.15	(5.5)
Decision authority	34.51	(6.4)			34.37	(5.9)	35.60	(7.2)			34.94	(7.4)
Skill discretion	38.77	(4.4)			38.31	(4.0)	38.26	(4.9)			37.21	(4.4)
Support from supervisor	10.95	(2.2)			10.61	(2.1)	11.68	(2.3)			11.71	(2.7)
Co-worker support	12.51	(1.7)			12.22	(1.5)	12.68	(1.8)			12.70	(1.5)
Perceived work attitude	72.22	(14.0)			27.49	(13.5)	70.00	(13.7)			68.30	(14.0)
Self-efficacy	67.01	(6.6)			72.03	(13.0)	66.16	(8.9)			64.70	(10.5)
Willingness to exert effort in completing the behaviour	25.98	(3.2)			25.11	(3.2)	25.44	(3.6)			24.58	(4.4)
Persistence in the face of adversity	25.35	(2.9)			25.23	(2.9)	24.78	(3.6)			24.79	(3.7)
Willingness to initiate behaviour	15.67	(2.8)			15.48	(2.8)	15.93	(3.1)			15.34	(3.4)
Work engagement total	4.41	(0.9)			4.50	(0.9)	4.29	(1.1)			4.44	(0.8)
Vigour	4.55	(0.9)			4.55	(1.0)	4.46	(1.0)			4.62	(0.9)
Dedication	4.76	(1.0)			4.84	(0.9)	4.70	(1.2)			4.77	(1.0)
Absorption	3.94	(1.2)			4.11	(1.1)	3.69	(1.2)			3.94	(0.9)

### Primary and secondary outcome measures

The results regarding the effectiveness of the intervention with respect to the primary and secondary outcome measures are presented in Table 3. A significant adverse effect during follow-up was found for work ability (B = −1.33, 95% CI −2.45 to −0.20) and vitality (OR = 0.10, 95% CI 0.02-0.46). This means that workers in the intervention group had a 1.33 points lower mean work ability score than workers in the business as usual group and they had a 0.10 times higher odds of being in a higher vitality category than the persons in the business as usual group. We found no statistically significant difference between the two groups for productivity. No interaction effects were found between the intervention and time to follow-up for any of the primary outcomes. Positive significant results in favour of the intervention group were found for the secondary outcomes work attitude (B = 5.29, 95% CI 0.99 to 9.59), self-efficacy (persistence subscale) (B = 1.45, 95% CI 0.43-2.48) and the skill discretion subscale from the job content questionnaire (B = 1.78, 95% CI 0.74-2.83).

**Table 3:** **Results of the regression analyses of the intervention on primary and secondary outcomes after 12 months**

**Outcome measure** ^**¥**^	**B**	**OR**	**95% CI**	
			**Lower**	**Upper**
**Primary outcomes** ^**+**^				
Work ability	−1.33		−2.45	−0.20
Vitality		0.10*	0.02	0.46
Productivity		0.83	0.23	3.00
**Secondary outcomes**				
Perceived fatigue	−0.11		−2.83	2.61
Job content				
Job demands	−0.82		−2.53	0.89
Decision authority	−0.55		−2.38	1.29
Skill discretion	1.78**		0.74	2.83
Support from supervisor	0.17		−0.59	0.94
Co-worker support	0.03		−0.41	0.46
Perceived work attitude	5.29*		0.99	9.59
Self-efficacy				
Willingness to exert effort in completing the behaviour	0.11		−0.95	1.17
Persistence in the face of adversity	1.45*		0.43	2.48
Willingness to initiate behaviour	−0.47		−1.33	0.40
Work engagement				
Vigour	−0.21		−0.47	0.05
Dedication	−0.03		−0.31	0.25
Absorption	−0.07		−0.40	0.26

^¥^Analyses on outcome measures were adjusted for age, gender, education, occupation, sector, shift work, duration current position and years paid work;^+^Primary outcomes were analysed with linear (work ability), logistic (productivity) and ordinal (vitality) multilevel analyses.

*p ≤ .05; **p ≤ .001.

### Process evaluation

All supervisors in the intervention group participated in the training before starting the intervention. The extent to which the intended components were provided to the workers in the intervention group (dose delivered) was evaluated by 54 (84%) workers in the process evaluation after three months. Almost all the workers (98%) received the information leaflets about the intervention and the booklet to prepare for their dialogue with their supervisors. A dialogue was planned for all workers.

The first stage of the intervention (inventory and modifiability) was performed by 91% (n = 49) of the workers. This stage supported 32 workers (65%) in formulating opportunities for personal development, career opportunities and problems experienced at work. Discussing amenability to change motivated 31 workers (63%) to plan actions.

The dialogue with the supervisor was held within three months after the baseline measurement for 52 (96%) workers. The average duration of the dialogue was 38 minutes (range 5–60 minutes) and the communication between workers and supervisor was generally good (n = 32; 62%) to very good (n = 17; 32%). Three workers required a follow-up meeting with their supervisors.

Preparation of the action plan in the third stage of the intervention (dose received) was performed by 29 workers (56%); 27 of these workers received feedback from their supervisor.

Satisfaction with the content and relevance of the intervention information, leaflets and booklet was reported by 53 workers (96%).

The intervention increased the ability of 44% of the workers (n = 23) to clarify and explore problems with work participation and career aspiration. About half the workers reported having become more capable at conducting dialogue with their supervisors about sustainable work participation (n = 25; 49%) and at setting up structured action plans to improve work conditions after the intervention (n = 23; 45%). Fifty-five percent (n = 29) of the workers stated that the intervention had made them aware of their responsibility in creating a healthy workplace. The intervention contributed to more self-confidence in changing the work situation for 39% (n = 20) of workers and to enhanced capability for discussing work performance with supervisors in 37% (n = 19). To a lesser extent, the intervention resulted in better functioning at work (n = 8; 16%) and more pleasure in the work environment (n = 10; 19%). The same trends were observed after nine months (Table 4).

**Table 4:** **Results of the process evaluation at workers’ level after 3 and 9 months**

	**After 3 months**			**After 9 months**		
	**N**	**%**	**N total**	**N**	**%**	**N total**
**The intervention has increased my ability to:**						
…clarify and explore problems with work participation and career aspirations	23	44	52	19	40	48
…establish a dialogue with the supervisor about sustainable work participation	25	49	51	24	50	48
…set up a structured plan to improve the work conditions	23	45	51	19	39	49
…perform my work as before	13	27	48	7	15	46
**Through the intervention I am:**						
…more aware of the responsibility to create a healthy and motivating workplace	29	55	53	28	60	47
…functioning better at work	8	16	51	7	15	47
…taking more actions to improve the work conditions	23	43	53	17	35	48
**The intervention contributed to:**						
…more pleasure in the work situation	10	19	52	8	17	47
…more self-confidence to accomplish changes in the work situation	20	39	51	14	30	47
…better skills to discuss work functioning with the supervisor	19	37	51	13	28	47

## Discussion

The ‘staying healthy at work’ problem-solving intervention designed for workers to enhance their sustainable employability showed no superior effect on productivity and a negative effect on work ability and vitality compared to business as usual. However, positive effects were found on the perceived work attitude secondary outcome measure, the persistence in the face of adversity self-efficacy subscale and the skill discretion subscale of the psychosocial work characteristics.

To our knowledge, this study is the first prospective controlled trial aimed at supporting the sustainable employability of ageing workers by means of an intervention focusing on enhancing the problem-solving capacity of workers. Most intervention studies aimed at promoting and enhancing participation in the workers’ working lives provide a lifestyle training programme to improve job retention, increase vitality or to decrease work disability [42-44]. This study provides workers with guidance on how to prolong work participation in good health by enhancing the workers’ awareness and behaviour by emphasizing their own decisive role in attaining goals and carrying out the necessary actions. As the labour participation of specifically ageing workers needs to be extended, the results of this study are innovative and provide valuable information for occupational health researchers, policymakers and employers. The fact that we found no or negative effects on the primary health-related outcomes is in line with earlier studies focusing on work-site interventions to support sustainable employability [45,46]. We have used Kristensen’s theoretical model to discuss our results [47]. Kristensen’s model allows us to distinguish between theory and programme failure (intervention being implemented but not effective versus the intervention being ineffective because it was not adequately implemented) and provide a systematic overview of the theoretical, methodological and practical issues of occupational intervention research.

Based on the results of previous problem-solving intervention studies we presume that our negative findings on the primary outcomes variables do not result from theory failure. These studies showed that problem-solving intervention studies on work-related outcomes had a superior effect on sickness absence [25], return to work [25,48], prevention of depression [49] and treatment of anxiety disorders [50]. Moreover, it has also been shown that problem-solving training in the workplace can increase problem-solving skills and problem-solving self-efficacy in the course of improving positive affect, job satisfaction and life satisfaction [51]. Therefore, our assumption that a self-directed cognitive behavioural intervention enhanced the problem-solving capacity of ageing workers towards sustainable employability could be effective remains valid. The positive results in our study on the secondary outcomes perceived work attitude, persistence in the face of adversity and skill discretion acknowledge that the intervention changed the awareness and behaviour of workers to enhance work participation. In addition, the process evaluation at the worker level confirms this. Therefore, we assume that the intervention’s lack of impact on the primary outcome measures must be explained by programme failure [47]. We will elaborate on both aspects of programme failure: dose delivered and dose received.

At the dose delivered level, the short duration of the training of the supervisors could explain the lack of effectiveness of the intervention on the primary outcome measures. The supervisors’ knowledge and basic skills in communication was low and, during the training, a lot of time was spent on these basic skills instead of on the problem-solving approach. Moreover, the duration and frequency of the training for supervisors (two sessions of two and five hours respectively) may have been too little to transfer the skills acquired into attitude and practice. Prior to implementation, the research team suggested training the supervisors for two days or three training sessions to accomplish the level of knowledge and skills necessary to perform the intervention. However, the intervention was conducted as part of the normal activities of an organization and the supervisors’ time to participate in the study was therefore limited. At the organizational level, management were convinced that their supervisors were trained well and the supervisors’ skills were in line with the skills necessary to perform the dialogue. Therefore, we had to deal with the restrictions to the supervisor’s time made available for training.

At the workers’ level (dose received), the extent to which the workers actively engaged in the third stage of the intervention was lower than expected. Whereas the number of intended intervention components actually delivered were positively evaluated and the intervention was implemented mostly as planned, 44% of the workers did not define a structured action plan after the dialogue. This low adherence may have contributed to the intervention’s low effectiveness. Based on reasons received by supervisors for not defining an action plan, reasons for drop-out during follow-up and written comments at the end of the questionnaire we assume that action plans were not defined because the workers were unable to translate the points discussed during the dialogue into appropriate actions, and due to high work pressure and lack of knowledge about available interventions in the organisation or lack of motivation. In contrast, the first step of the intervention was performed by almost all workers. This discrepancy in adherence between parts of the intervention could also partly explain our findings. The first step makes workers aware of their own responsibility for, obstacles with, and retention factors and needs for sustainable employability. As a consequence, workers can experience feelings of increased workload and decreased work ability. Most positive experiences and effects result from the interventions subsequent solution-focused steps. These steps were not followed by many workers and, if followed, their effect appears later. The positive results on the secondary outcomes in our study imply that the intervention improved the workers perspectives on awareness of and responsibility for sustainable employability. These results are in line with the results of other behavioural intervention studies which have shown that cognitive behavioural interventions improve self-efficacy, job satisfaction and motivation to return to work [42,52] and to coping with chronic conditions [53,54]. Workers in our intervention understood their own responsibility towards a healthy and sustainable working life, but missed the optimal benefit of the intervention’s later steps. Therefore we assume that the intervention could be effective over the long term in enhancing sustainable employability if programme failures are avoided.

In addition to the time frame for the intervention, which is discussed above in relation to programme failures, the non-randomized design might have caused bias. The earlier the supervisor agreed to participate, the more likely it was that he/she was allocated to the intervention group. The departments in the intervention group were comparable to the business as usual group because the departments asked to participate in the study were matched on their proportion of ageing workers and comparable job tasks to overcome this source of bias in the study design. However, it is possible that especially cooperative and committed supervisors who already put a lot of effort into the sustainable employability of their ageing workers or supervisors experiencing problems in supporting the needs of their workers to continue their working lives on their own were allocated preferentially to the intervention. Contamination between supervisors in the business as usual and intervention group cannot be excluded. Although we applied cluster randomization to avoid contamination between workers, supervisors from the two groups may have had common activities within the organization and could have discussed the study. Furthermore, there was a small difference between the intervention and the business as usual groups at baseline for the job demands secondary outcome measure. However, this and other differences between the two groups were taken into account in the analyses.

Despite the abovementioned shortcomings, our intervention study also had some successes. The results suggest that workers in the intervention group were better able to identify or discover effective solutions for the specific problem encountered in working life compared to workers in the business as usual group who received an annual appraisal. Although both groups of workers receive an annual appraisal, the results shows that the intervention stimulated workers to be aware of their own responsibility to create a healthy and motivating workplace. This is in line with experiences of management from the participating organizations, because in practice most workers feel that they are being judged and marked on performance. Workers do not discuss what they enjoy doing, where they have difficulties, aspirations and opportunities because they do not know how to prepare beforehand. Moreover, a strength of the intervention was its integrated approach, providing close collaboration between the worker and supervisor as well as human resource management, which ensures that employees have the tools, resources, training and development needed to carry out their responsibilities successfully. Earlier research has shown that a more participatory and supportive approach from the supervisors could help workers identify their challenges and implement solutions [55]. The close cooperation with the human resource professionals during the implementation ensured that the intervention was in line with the existing organizational policy. However, during implementation it is important consider fidelity (delivering the intervention as planned), because non-fidelity dilutes the difference between the intervention and the business as usual group. An additional asset of the intervention is that the method can be incorporated into the annual appraisal cycle between worker and supervisor within the organizations.

Future research into intervention studies at workplaces based on an integrated problem-solving approach to enhance sustainable employability needs to be carried out with strict programme integrity – with a detailed process evaluation at both the worker and the supervisor levels, and follow-up over a long period (>1 year) – to establish whether the intervention is effective in increasing sustainable employability. What is needed now is a randomized controlled trial including a detailed process evaluation at both the worker and supervisor levels to evaluate the effectiveness of the intervention. Programme failures could be avoided by a) development of a monitoring system to support the establishment of a structured plan after dialogue with specific, well-defined and realistic goals, and actually to perform the actions and solutions described in this plan, and b) telephone support and the organization of peer groups for supervisors during the intervention period to improve their communication skills and support role based on the problem-solving approach. In addition, information about the quality of the annual appraisal and the support received from supervisors in the business as usual group could show the extent to which the intervention contributes to a healthy working life compared to the traditional appraisal. In addition, it is important to investigate the usability and effectiveness of the intervention in different occupational groups and/or professions, and how different types of chronic health conditions affect the effectiveness of the intervention.

## Conclusion

Given an ageing workforce, evidence-based interventions are needed to prepare workers for the prospects of working longer. The ‘staying healthy at work’ intervention provides a self-directed cognitive behavioural strategy to enhance the problem-solving capacity of ageing workers to help them achieve sustainable employability, whereas most interventions to extend working life are based on promoting workers’ health. The intervention seemed to result in beneficial effects on perceived work attitude, self-efficacy and skill discretion, but showed no effects on work ability, vitality and productivity compared to business as usual.

## Abbreviations

HRP(s): Human Resource Professional(s)
WAI: Work Ability Index
SF12: 12-Item Short Form Health Survey
QQ: Quality and Quantity
CIS: Checklist of Individual Strength
JCQ: Job Content Questionnaire
WIS-DLV: Dutch Language version of the Work Involvement Scale
ALCOS-16: Dutch version of the General Self-Efficacy Scale
UWES-9: Dutch version of the Utrecht Work Engagement Scale
